# The Interaction between Phagocytes and *Streptococcus agalactiae* (GBS) Mediated by the Activated Complement System is the Key to GBS Inducing Acute Bacterial Meningitis of Tilapia

**DOI:** 10.3390/ani9100818

**Published:** 2019-10-16

**Authors:** Yu Liu, Liping Li, Ting Huang, Wende Wu, Wanwen Liang, Ming Chen

**Affiliations:** 1 Guangxi Academy of Fishery Sciences, Fish diseases control and prevention lab, Qingshan Road NO.8, Nanning 530021, China; liuyudennil@163.com (Y.L.); pinglili2000@163.com (L.L.); htwish@163.com (T.H.); nnlww@163.com (W.L.); 2 Guangxi University, Daxuedong Road NO.100, Nanning 530004, China; wwd0194@163.com

**Keywords:** streptococcicosis, *Oreochromis*, meningitis, duel RNA-seq, proteome, vaccine

## Abstract

**Simple Summary:**

*Streptococcus agalactiae* (GBS) is a serious threat to farmed tilapia, which results in high mortality and seriously hinders tilapia farming development. The pathogenic mechanism of tilapia infected with GBS which die rapidly in production remains unknown. We provided a comprehensive comparative analysis of the tilapias infected with fish-derived GBS attenuated strain YM001 and its parental virulent strain HN016. The present study indicates that the interaction between phagocytes and GBS mediated by the activated complement system is key to GBS inducing tilapia acute bacterial meningitis. The low survival ability caused by reduced β-lactam antibiotics resistance is one of the important reasons YM001 lost its pathogenicity to tilapia. Our study provided a comprehensive cognition of the mechanism of acute bacterial meningitis caused by GBS.

**Abstract:**

*Streptococcus agalactiae* is an important pathogen for tilapia meningitis. Most of the infected tilapia die rapidly in production, when the way to study the pathogenic mechanism of bacteria on host through chronic infection in laboratory is not comprehensive and accurate enough to elucidate the real pathogenic mechanism. The objective of this study was to investigate the mechanism of acute bacterial meningitis of tilapia caused by *Streptococcus agalactiae* (GBS), and provide a theoretical basis for its prevention and treatment. Duel RNA-seq, proteome analysis, histopathological analysis, plasma biochemical indexes, and blood routine examination were performed on tilapias infected with fish-derived GBS attenuated strain YM001 and its parental virulent strain HN016. The results showed that the contents of white blood cell (WBC), monocytes (MON), and neutrophil (NEU) were significantly lower in the HN016 group compared to that in the YM001 group (*p* < 0.05). Histopathological examination showed that there were partially lesions in the examined tissues of tilapia infected by HN016, while no obvious histopathological changes occurred in the YM001 group. The differential expressed genes (DEGs) and differential expressed proteins (DEPs) between YM001 and HN016 were mainly enriched in the beta-lactam resistance pathway (oppA1, oppA2, oppB, oppC, oppD, oppF, and mrcA). The DEGs DEPs between YM001-brain and HN016-brain were mainly enriched in the complement and coagulation cascades signaling pathway (C2a, c4b, c3b, c7, CD59, ITGB2, and ITGAX). The present study indicates that the interaction between phagocytes and GBS mediated by the activated complement system is the key to GBS inducing tilapia acute bacterial meningitis. The low survival ability caused by reduced β-lactam antibiotics resistance is one of the important reasons for why YM001 lost its pathogenicity to tilapia.

## 1. Introduction

With the fast development of aquaculture, large-scale farming has become the main production mode in fish farming. Simultaneously, high density farming leads to high incidence of diseases such as streptococcosis, which results in high mortality and seriously hinders tilapia farming development. Since 2009, large-scale streptococcal epidemics have occurred continuously in the Chinese tilapia aquaculture industry with a mortality rate of 30% to 90%, and more than 90% of the clinical strains are *Streptococcus agalactiae* (GBS) [1,2,3,4]. Streptococcal meningitis caused by GBS is one of the main causes of death when farming tilapia [5,6,7]. Thus, understanding pathogenic mechanism of bacterial meningitis enables great achievements in the prevention, control, and treatment of GBS infection.

In vitro and in vivo studies have previously confirmed that both GBS virulence factors and host immune responses contribute significantly to the development of bacterial meningitis. Microbial invasion and traversal of the blood-brain barrier (BBB) is a prerequisite for meningitis and the associated BBB dysfunction [8]. As the blood-borne pathogen, *S. agalactiae* must interact with cerebral microvascular endothelial cells (BMECs) which constitute the bloodebrain barrier, while subsequent bacterial replication within the central nervous system (CNS) provokes host inflammatory response, resulting in meningitis [9]. It has been reported that GBS could be internalized by marine macrophages and survive intracellularly for more than 24 h, inducing injury to macrophages [10]. Afterwards, the macrophages serve as vectors for the blood-borne spread of GBS to cerebral endothelial cells, followed by further spread into the brain parenchyma [11,12,13]. Pathogens can cross the BBB transcellularly in infected phagocytes. The intracellular localization of GBS in macrophages may protect the organism from more active antimicrobial molecules and spread in the blood [9]. Several specific GBS virulence factors had been identified by in vitro and in vivo models. These factors, such as laminin-binding protein (Lmb), can promote the invasion ability of the GBS towards human brain microvascular endothelial cells [9]. The fibrinogen-binding protein encoding gene fbsB plays a crucial role in the invasion of *S. agalactiae* into human epithelial cells [14]. C5a peptidase is a bifunctional protein, which enzymatically cleaves C5a and mediates adherence to a host molecule and evasion of the host immune system [15]. The BBB invasion by GBS depends upon proper cell-surface anchoring of lipoteichoic acid [16]. However, these studies are insufficient for understanding the mechanism of meningitis in tilapia caused by GBS mainly due to two reasons. First, although the GBS strains of human and fish sources contain similar virulence factors [17,18], some studies have shown that there are differences in the virulence mechanisms between human and fish GBS [17,19]. Second, most of the infected tilapia die rapidly in production when the way to study the pathogenic mechanism of pathogenic bacteria on host through chronic infection in laboratory is not comprehensive and accurate enough to elucidate the real pathogenic mechanism. Therefore, a better method is needed to study the mechanism by which tilapia infected with GBS causes rapid death from acute meningitis.

We have previously obtained a fish-derived attenuated GBS strain YM001 (Ia,ST7) via continuous passage in vitro from its parental strain HN016 (Ia,ST7). HN016 can induce typical meningitis symptoms while YM001 has no pathogenicity to tilapia. YM001 has good immunogenicity and can provide good immune protection to tilapia [20]. Genome study shown that the YM001 had two large deletions (D1, 5832 bp; D2, 11,116 bp) compared with HN016 [18]. The two phenomena of acute death of tilapia bacterial meningitis induced by GBS and immune protection induced by GBS attenuated strain can be fully simulated when tilapia are treated with HN016 and YM001, respectively. Combined duel-RNA seq and proteome analysis can provided a comprehensive understanding of the key pathogenic mechanism of GBS causing acute meningitis in tilapia. This study provides a good theoretical basis for the subsequent research and development of relevant vaccines, and also provides a certain theoretical basis for the study of human GBS bacterial meningitis.

## 2. Materials and Methods

### 2.1. Bacterial Strains and Fishes

*S. agalactiae* strain HN016 was isolated from a moribund cultured tilapia with typical clinical and pathogenic characteristics of meningitis (Hainan, China, 2010), belonged to GBS serotype Ia, multilocus sequence type seven (ST7). The HN016 strain was used as the starting material to generate an attenuated strain by continuous passage in vitro, and the 840th passage was named strain YM001 (Ia,ST7) [20].

Non-infected Nile tilapia (*Oreochromis niloticus*) with average weight of 400 ± 15.10 g were provided by the National Tilapia Seed Farm (Nanning, Guangxi, China), which were confirmed to be negative for bacterial infection by bacteriological analysis of the brain and kidney samples. The fishes were monitored twice a day with a formulated diet (Tongwei Feed Company, Nanning, China).

### 2.2. Ethics Statement

Animal experiments were conducted in strict accordance with the Chinese animal experiment ethical inspection, under project license number: GXU2015039, approved by the Guangxi University, China.

### 2.3. Challenge and Behavioral Assessments

The strains were cultured following our previous methods [20]. Briefly, the strain HN016 and YM001 were removed from a −80 °C refrigerator, then streaked onto a 5% sheep blood agar plate, and cultured at 30 °C for 24 h. A single colony was then picked up, inoculated into 10 mL of TSB medium, and cultivated at 30 °C by shaking. After 12 h, 1.0 mL of bacteria was inoculated into fresh 10 mL of TSB medium and cultured continuously by shaking for another 12 h. The bacterial density (CFU mL^−1^) was determined by plating 100 µL of 10-fold serially-diluted culture onto sheep blood agar plates and counting of the colonies.

After anesthesia by immersion into a bath of 10 mg L^−1^ benzocaine (Sigma, St. Louis, USA), a total of 60 fishes were equally divided into three groups, each group with two tanks (10 fishes/tank, with 2 replicates). Three groups were intraperitoneally injected with 0.1 mL concentrated YM001 (1.0 × 10^10^ CFU mL^−1^), HN016 (1.0 × 10^10^ CFU mL^−1^), and sterile TSB, respectively. The infected fishes were monitored every 0.5 h and raised in fiber reinforced plastic tanks at 33 ± 1 °C. When the HN016 group passed away, we sacrificed all the fishes in the three groups and sampled the brain tissues, which were stored in the −80 °C refrigerator. Meantime, the bacteria were isolated and identified from the brain and spleen tissues.

### 2.4. Blood Sampling

For each individual, 1.0 mL blood from caudal vein was collected. In total, six individuals from each group were contained. The blood samples were divided into two parts. One was treated using sodium citrate to obtain anticoagulanted whole blood and one was stored at room temperature for 2 h to collect upper serum. The anticoagulanted whole blood was used to assay routine blood test by using URIT-5160 vet (Mindray, China) and upper serum was used to analyze biochemical indexes using H7600 (HITACHI, Japan). Significant differences among the groups were identified by one-way analysis of variance (ANOVA) using SPSS 17.0 software (SPSS Inc., Chicago, IL, USA). *p* < 0.05 was determined as significant.

### 2.5. Tissue Injury Pathological Analysis

After blood sampling, the fishes were sacrificed with high concentration of benzocaine before the brains were collected. Following standard fixation in 10% neutral buffered formalin and sample processing in paraffin wax blocks, paraffin sections (6 μm thick) were stained with hematoxylin and eosin (H&E) for light microscopy observations.

### 2.6. Dual RNA-seq Transcriptional Analysis

Dual RNA-seq methods refers to Alexander et al. [21], with minor modifications. Briefly, stored brain tissue and strains were sent to the Majorbio Co., LTD (Shanghai, China) for duel-RNA saq and proteome analysis. The total RNAs were isolated using TRIzol reagent (Invitrogen, Carlsbad, CA, USA) according to the manufacturer’s instructions. The concentrations and concentration and purity of total RNAs were analyzed using Nanodrop 2000. The quality of RNA was determined using Agilent 2100 Bioanalyzer (Agilent Technologies, Santa Clara, CA, USA). Then, the total RNAs were examined following rRNA removing by a Ribo-Zero rRNA Removal Kit (Epicentre, Madison, WI, USA) and fragmented into ~300 bp long fragments. Illumina TruseqTM RNA sample prep Kit was employed to prepare the cDNA libraries according to the manufacturer’s instructions. Subsequently, the sequencing of cDNA libraries was performed on an Illumina HiSeq4000 sequencing platform.

Raw sequences were first filtered. The reads with N ratio >10% or length <20 bp were discarded. The clean reads were mapped to tilapia genome and *S. agalactiae* strain genome by Hiast2 (http://ccb.jhu.edu/software/hisat2/index.shtml) [22] respectively. Gene expression was measured by (Fragments Per Kilobase of exon model per Million mapped reads) FPKM values using RSEM (http://deweylab.github.io/RSEM/) [23]. The genes with |log2FC| ≥ 1 and FDR <0.05 were determined as differentially expressed genes between two groups. Functions of differentially expressed genes were defined by KEGG pathways (http://www.genome.jp/kegg/). *p* < 0.05 was confirmed as an enrichment pathway. The enrichment analysis of KEGG pathway was performed by KOBAS (http://kobas.cbi.pku.edu.cn/home.do) [24,25].

Raw data had been submitted to National Center for Biotechnology Information (NCBI) with the accession numbers <SRX6434250>, <SRX6434249>, <SRX6427738> and <SRX6433682> for YM001, HN016, YM001-brain, and HN016-brain, respectively.

### 2.7. TMT Protein Labeling and LC–MS/MS Proteomic Analyses

Brain tissues (~100 mg for each sample) were homogenized and decomposed by 1 mL lysis buffer containing 8 M urea, 1% SDS abd 65 mM dithiothreitol on ice for 30 min. After 2 min break by ultrasonic processor, the total proteins were isolated by centrifuging at 4 °C, 12,000 *g* for 20 min. The protein concentrations of supernatant were determined by BCA method and the qualities of protein were assayed by 12% SDS-PAGE analysis. After that, 100 μg protein of each samples were performed reductive alkylation with 10 mM TCEP at 37 °C for 60 min and 40 mM Iodoacetamide at room temperature for 40 min. Subsequently, the samples were digested with trypsin at 37 °C overnight. The TMT protein labeling was conducted using a Six-plex TMT labeling kit (Thermo Scientific, Shanghai, China) following the manufacturer’s instructions. Briefly, one tube of TMT was added to 100 μg digested protein and incubated for 2 h at room temperature after being incubated with hydroxylamine for 15 min at room temperature. Samples were dissolved in UPLC buffer and separated using high pH reverse-phase HPLC coupled with Agilent 300 Extend C18 HPLC column. A gradient of 2% and 80% acetonitrile in ammonium bicarbonate (pH = 10) was applied to fractionate peptides with speed of 200 μL/min over 66 min. The peptides were divided into 20 fractions and dried by vacuum centrifugation. Then, the second dimensional liquid chromatography was analyzed. LC–MS/MS analysis was performed using C18 column (75 μm × 25 μm) on EASY-nLC200 and Q-Exactive. The constant flow rate was 300 nL/min for 120 min. Solvent A and solvent B was 2% ACN with 0.1% formic acid and 80% ACN with 0.1% formic acid, respectively. For MS scanning, the m/z scan range was 350 to 1300. The resolution was 70,000 and 35,000 for the two degrees scanning.

The resulting data were collected by Thermo Xcalibur 4.0. We used Proteome DiscovererTM Software 2.1 to identify proteins from NCBI nr and SwissProt/UniProt with default parameters. The protein was assigned to gene ontology (GO) and KEGG categories. Differentially expressed proteins were identified by T-test. When *p* < 0.05 and fold change (FC) fulfilled as FC <0.83 or FC >1.20, the proteins were determined as differentially expressed proteins. KEGG enrichment of differentially expressed proteins was differentiated when adjusted *p* < 0.05.

The mass spectrometry proteomics data have been deposited to the ProteomeXchange Consortium via the MASSIVE with the dataset identifier <PXD014616>.

### 2.8. Validation of Gene Expression Level by Real-Time Quantitative PCR (RT-qPCR)

The RT-qPCR was used to verify the expression levels of the candidate genes between different strains and infected brain tissues. The recA [26] and β-actin [27] gene were selected as the standardization controls. The specific primers used to amplify the candidate genes were designed using Primer 5 software. Briefly, total RNA was extracted from HN016, YM001 HN016-brain, and YM001-brain. Then it was reverse transcribed into cDNA by HiScript^®^ II 1st Strand cDNA Synthesis Kit (+gDNA wiper) (Vazyme, Nanjing, China). Real-time qPCR was performed in a DNA Engine Chromo 4 real-time system (BioRad) with HiScript II One Step qRT-PCR SYBR Green Kit (Vazyme, Nanjing, China). The expression of genes was calculated as relative expression to recA using the 2(−Δ Δ C(T)) method and samples were analyzed in triplicates.

## 3. Results

### 3.1. Overviews of RNA Transcriptomic and Quantitative Proteomic Analyses Profiles

**Figure 1:**
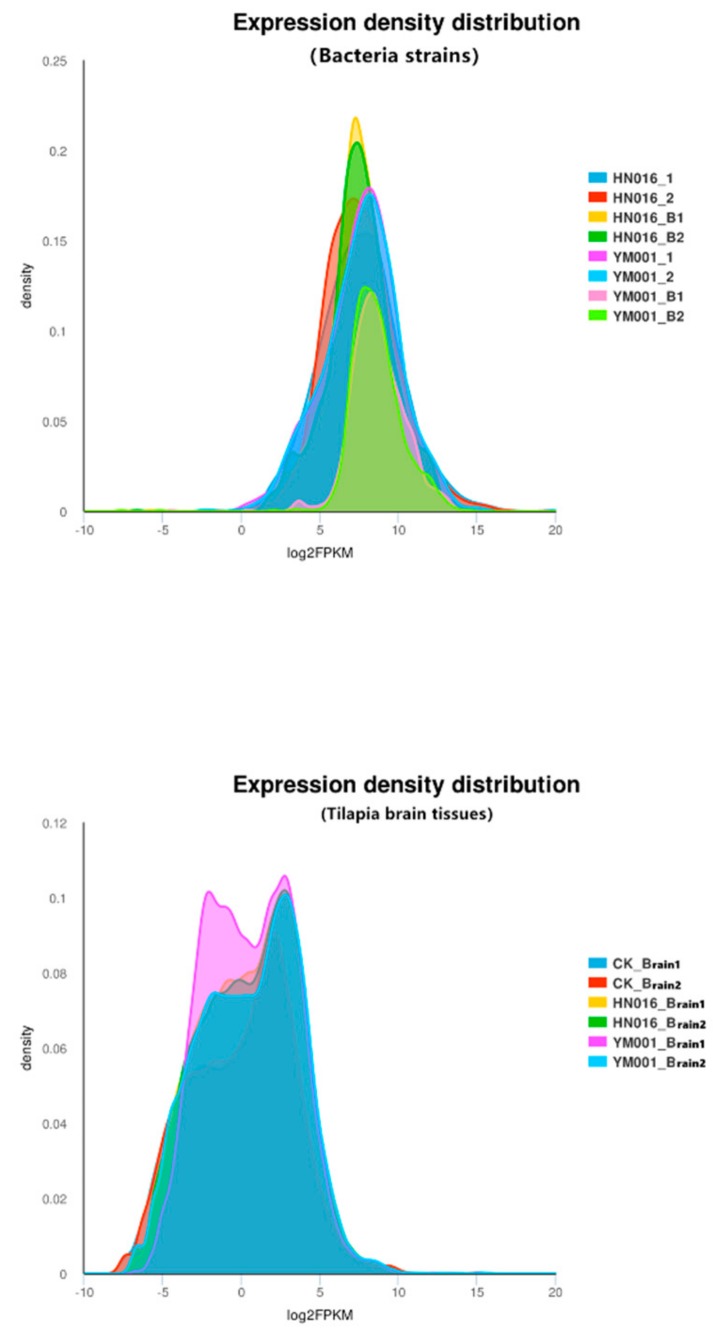
Expression density distributions of bacteria and brain tissues.

In duel RNA-seq transcriptomic analyses, a total of 81,117,428,312 raw reads [2,756,488,088 and 2,809,282,218 for YM001, 2,451,870,654 and 2,488,122,734 for HN016, 7,756,265,396 and 7,979,039,320 for CK-brain (blank control), 13,242,751,340 and 14,067,560,754 for HN016-brain, as well as 12,669,872,138 and 14,896,175,670 for YM001-brain, respectively] were generated, and 71,687,393,515 clean reads were obtained after cleaning and quality checks. Approximately 97.52% (96.30%–98.82%) of the prokaryotic reads and 88.44% (87.32–90.14%) of the eukaryotic reads acquired from the RNA-seq experiment involved mapping to the reference genome (Figure 1). A total of 1486 genes (1048 up-regulated and 438 down-regulated) and 33 proteins (33 up-regulated and 0 down-regulated) were significantly altered (*p* < 0.05) in YM001 vs. the HN016 group. 29,275 genes (10,632 up-regulated and 18,643 down-regulated) and 79 proteins (12 up-regulated and 67 down-regulated) were significantly altered (*p* < 0.05) in YM001-brain vs. the HN016-brain group.

### 3.2. Histopathological Analysis

**Figure 2:**
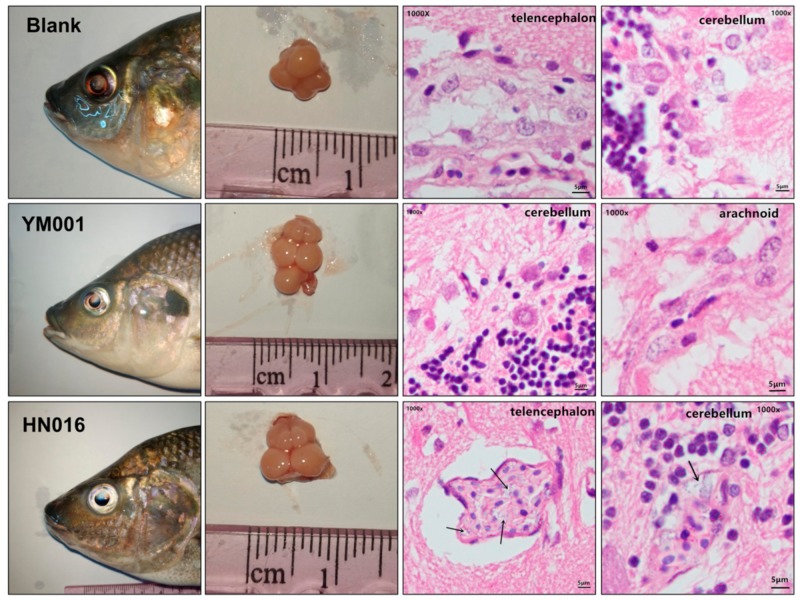
Necropsy and pathological sections of the tilapia brain tissue. Black arrows indicate a large number of visible blue dye-stained Streptococcus particles in the HN016 group, which are hardly seen in the blank group and the YM001 group.

The brain tissues of the HN016 group, YM001 group, and blank group showed no significant difference during gross observation. Histopathological examination showed that there were partial lesions in the examined tissues of tilapia infected by HN016, which included edema, loose and thickening in meninges, disperse from brain matrix, interstitial inflammatory cell infiltration, capillary congestion, bleeding, and a lot of visible blue dye-stained Streptococcus particles. In contrast, no obvious histopathological changes were observed in fish injected with YM001 and in the control group (Figure 2).

### 3.3. Effects of HN016 and YM001 on Blood Parameters of Tilapia

**Figure 3:**
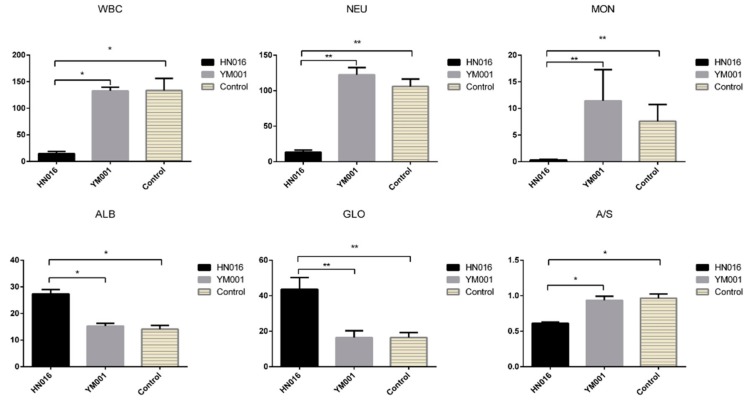
Plasma biochemical indexes and blood routine examination of the experimental tilapias. The results show the significant difference of the blood parameters in the tilapias between the YM001 group and the HN016 group.

Plasma biochemical indexes and blood routine examination were assayed to show the differences after infection with low and high virulence strains. The contents of white blood cell (WBC), monocytes (MON), and neutrophil (NEU) were significantly lower in the HN016 group compared to that in the YM001 group (*p* < 0.05) while no significant differences were found between the control and the YM001 group. The contents of albumin (ALB) and globulin (GLO) were significantly higher in the HN016 group compared to that in the YM001 group (*p* < 0.05) while no significant differences were found between the control and the YM001 group. In contrast, the ALB/GLO (A/G) ratio was the lowest in HN016 compared to in other groups (*p* < 0.05) (Figure 3).

### 3.4. Bioinformatics Analysis of DEGs and DEPs

For the bacteria strains, all differentially expressed mRNA and proteins had their functions classified by using GO and KEGG databases. The DEGs between YM001 and HN016 were enriched in different signaling pathways, such as the phosphotransferase system (PTS), propanoate metabolism, amino sugar and nucleotide sugar metabolism, and beta-Lactam resistance.

**Figure 4:**
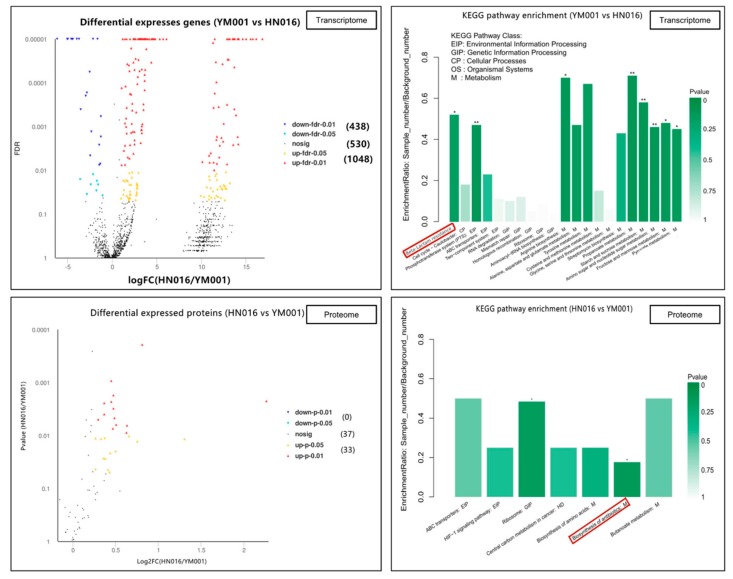
Bioinformatics analysis of differential expressed genes (DEGs) and differential expressed proteins (DEPs) between YM001 and HN016 strains. Multi-omics analysis show the main differences between the YM001 strain and HN016 strain.

**Figure 5:**
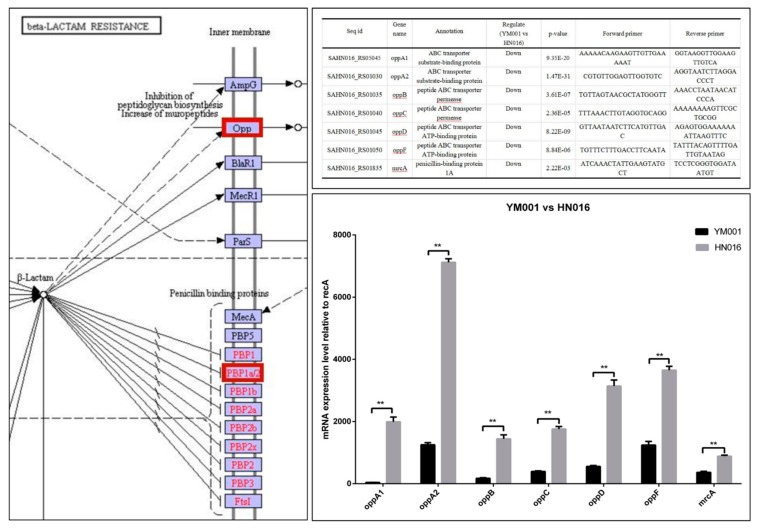
RT-qPCR verification. Bioinformatics analysis shows that the beta-lactam resistance signaling pathway may play an important role in virulence. RT-qPCR shows that the candidate genes have the same tendency, which further verified the reliability of the multi-omics analysis results.

The DEPs YM001 and HN016 were mainly enriched in two signaling pathways: biosynthesis of antibiotics and ribosome (Figure 4). The Beta-Lactam resistance pathway may play an important role in the virulence attenuation in YM001 (Figure 4). The candidate genes in β-Lactam resistance pathway were verified by qRT-PCR (Figure 5).

**Figure 6:**
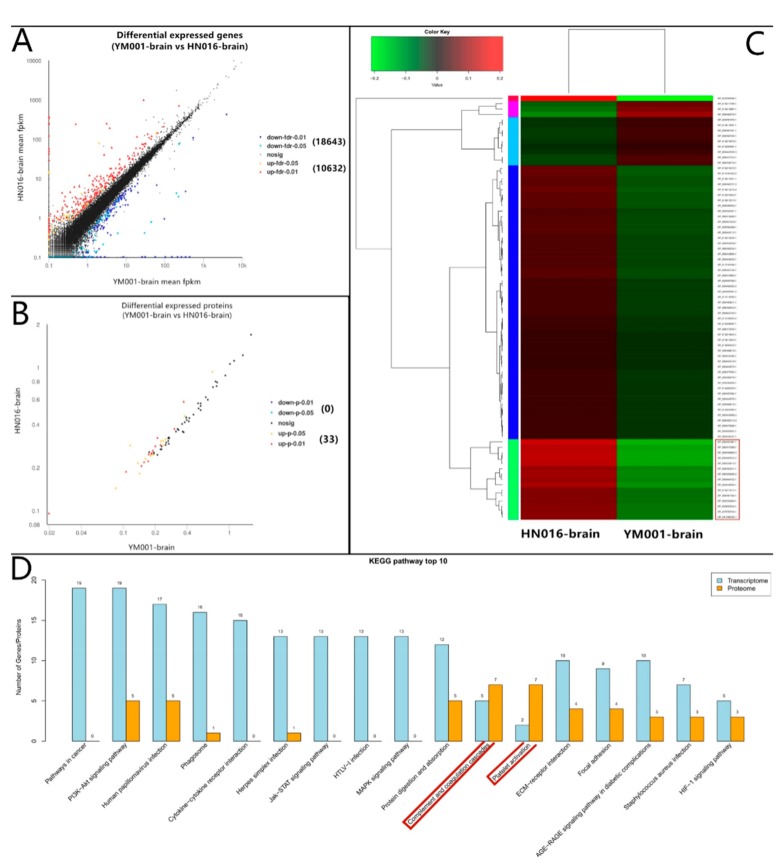
Bioinformatics analysis of DEGs and DEPs between YM001-brain and HN016-brain. (**A**) shows DEGs between YM001-brain and HN016-brain. (**B**) shows the DEPs between YM001-brain and HN016-brain. (**C**) shows the significant DEPs cluster analysis heat map between YM001-brain and HN016-brain. (**D**) shows the most enriched KEGG pathways between YM001-brain and HN016-brain. The complement and coagulation cascades signaling pathway may plays an important role in virulence.

**Figure 7:**
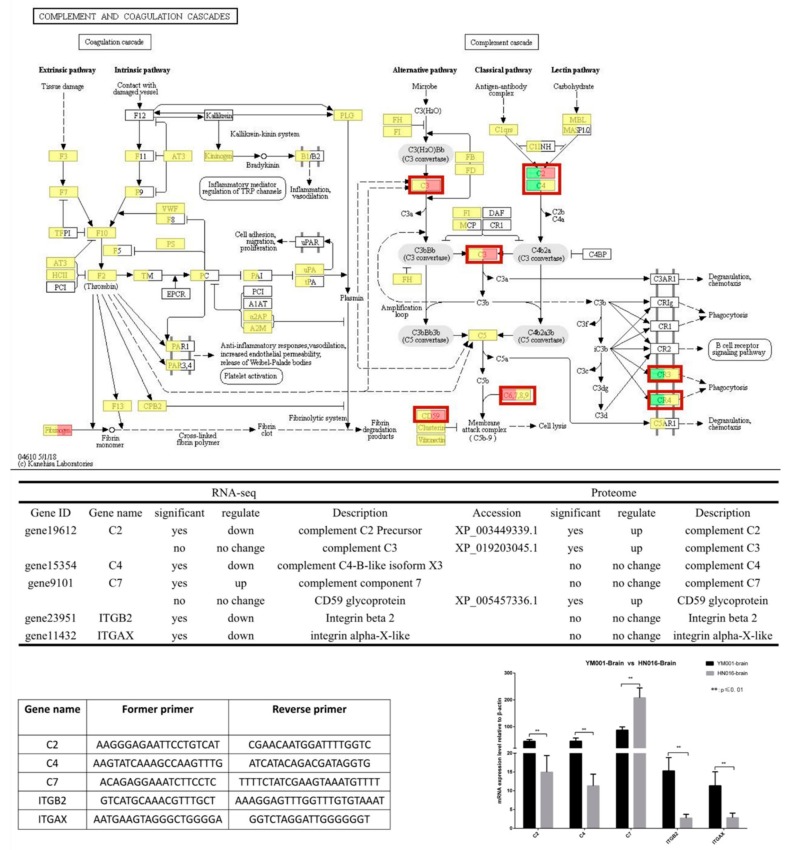
The complement and coagulation cascades signaling pathway and RT-qPCR verification. Bioinformatics analysis showed the significantly differential genes and proteins in the complement and coagulation cascades pathway between the YM001-Brain and HN016-brain. RT-qPCR showed that the candidate genes have the same tendency, which further verified the reliability of the multi-omics analysis results.

For tilapis brain tissues, all differentially expressed mRNA and proteins were used to classify the functions with the GO and KEGG databases (Table 1). The DEGs between YM001 and HN016 were mainly enriched in two signaling pathways: complement and coagulation cascades and Platelet activation (Figure 6). Genes enriched in the complement and coagulation cascades signaling pathway (C2, C4, C7, ITGB2, and ITGAX) were identified as important candidate genes responsible for influencing the pathogenicity of GBS strain to tilapia. The candidate genes in complement and coagulation cascades signaling pathway were verified by qRT-PCR (Figure 7).

## 4. Discussion

RNA-seq provides a sensitive method for global gene expression analysis in infection biology [21]. Previous combined transcriptomic and proteomic studies on HN016 and its attenuated strain YM001 cultured in vitro have shown that the pentose phosphate pathway and pyruvate metabolism pathway that affecting the growth of the strain may be one of the important reasons for the virulence attenuation in HN016 [28]. However, as bacterial infections of eucaryon involve two interacting organisms, there are interactions between the bacteria and the host. Therefore, studies of cultured bacteria in vitro do not fully reveal the pathogenic mechanism of the GBS. A comprehensive understanding of host-pathogen interactions requires a knowledge of the associated gene expression changes in both the pathogen and the host [21]. Here, dual RNA-seq has been used to discover the differences between HN016 and YM001, and their consequences for the tilapia host response, which can avoid tedious and error-prone physical separation of pathogen and host.

Bacterial resistance refers to the resistance of bacteria to the action of antibacterial drugs. Resistant strains have a stronger ability to survive, threatening the health of the host. For the animal strains of the GBS, the phenomenon of the resistance to antibiotics is more often met than in human strains [29]. β-lactam antibiotics are one of the oldest and most prescribed classes of antibacterial treatments worldwide [30], which is widely used in GBS treatment [31,32,33,34]. We have shown that Opp (oppA1, oppA2, oppB, oppC, oppD and oppF) and PBP1a (mrcA) encoding genes were significant down-regulated in YM001, which were regulating the β-lactam antibiotics resistance of the GBS. The reduction of β-lactam antibiotics resistance will lead to the decrease of survivability of YM001. There was no significant difference of the eyes and brain tissues of tilapia between the three groups found by gross observation, but a large number of visible blue dye-stained Streptococcus particles were observed in brain sections in the HN016 group under the microscope which were hardly seen in the YM001 group. This indicates that the low survival ability caused by reduced β-lactam antibiotics resistance is one of the important reasons for YM001 to lose its pathogenicity to tilapia. OppA1, oppA2, oppB, oppC, oppD, oppF, and mrcA may be the most important target sites.

Complementing these sites is a group of proteins that exist in the body fluids and cell surfaces of animals which have biological activity after activation, which in turn can mediate immune and inflammatory responses [35]. C2a is the fragment of component C2, which combines with complement factor C4b to generate the C3 or C5 convertase [36,37,38]. C4b is the non-enzymatic component of the C3. C5, which convertases and is thus essential for the propagation of the classical complement pathway, covalently binds to immunoglobulins and immune complexes and enhances the solubilization of immune aggregates [39]. Subsequently, C3 cleaved into C3a and C3b by C3 convertase, activating C3b that can bind covalently, via its reactive thioester, to cell surface carbohydrates or immune aggregates [40]. Integrins ITGB2 contributes to natural killer cell cytotoxicity [41], and is involved in leukocyte adhesion and transmigration of leukocytes including T-cells and neutrophils [42,43]. Integrin ITGAL/ITGB2 in association with ICAM3, contributes to apoptotic neutrophil phagocytosis through macrophages [44]. ITGAX mediates cell-cell interaction during inflammatory responses. It is especially important in monocyte adhesion and chemotaxis [45]. We have shown that C2a and C4b were significantly down-regulated in YM001-brain, resulting in C3 being significantly up-regulated when compared with HN016-brain. Ultimately, lead Integrin ITGB2 and ITGAX are significantly down-regulated. It has been shown that GBS can survive for a long time after being phagocytosed by phagocytic cells such as macrophages and neutrophils [46,47,48]. As a result, it can escape the killing effect of active antibacterial molecules in the blood, which is the basis of GBS-induced bacteremia and subsequent meningitis [10]. The down-regulation of ITGB2 and ITGAX in YM001-brain leads to a decreased ability of YM001 to evade the killing effect of active antibacterial molecules in the blood by hiding in phagocytic cells such as macrophages and neutrophils, thus leading to reduced survivability. Therefore, YM001 lost its pathogenicity to tilapia.

C7 is a constituent of the membrane attack complex (MAC) that plays a key role in the innate and adaptive immune response by forming pores in the plasma membrane of target cells. CD59 is the potent inhibitor of the complement membrane attack complex (MAC) action [49]. Plasma biochemical indexes and blood routine examination shown that WBC, MON, NEU and A/S in HN016-infected group were significantly lower than the YM001-infected group and the control group. Meantime, CD59 was significantly up-regulated in YM001-brain when compared with HN016-brain and inhibited cell lysis. This suggests that in acute bacterial meningitis infection, GBS may cross the BBB transcellularly in infected phagocytes and reach the brain, subsequently destroying the phagocytes, leading to GBS being released and proliferating to induce meningitis.

## 5. Conclusions

In summary, the interaction between phagocytes and GBS mediated by the activated complement system is the key to GBS inducing tilapia acute bacterial meningitis. The low survival ability caused by reduced β-lactam antibiotics resistance is one of the important reasons why YM001 lost its pathogenicity to tilapia. C2a, c4b, c3b, c7, CD59, ITGB2, ITGAX, oppA1, oppA2, oppB, oppC, oppD, oppF, and mrcA may be the most important target sites for examination. Our study provided a comprehensive cognition of the mechanism of the acute bacterial meningitis caused by GBS.

## Figures and Tables

**Table 1:** Annotations of the significant DEPs between HN016-brain and YM001-brain.

Accession	Description	YM001-Brain Relative Expression Level	HN016-Brain Relative Expression Level
XP_003451887.1	collagen alpha-1(II) chain isoform X1	1.524	2.9755
XP_003439010.1	collagen alpha-1(II) chain isoform X1	1.332	2.451
XP_003438858.1	collagen alpha-3(IX) chain isoform X1	0.8685	1.4765
XP_005461453.1	collagen alpha-1(XI) chain	0.8435	1.2605
XP_005459969.3	vitellogenin	0.572	1.0565
XP_003452622.2	vitellogenin-2	0.545	1.0055
XP_003447299.1	apolipoprotein A-I	0.5	0.9025
XP_003453341.1	leukocyte cell-derived chemotaxin 1	1.387	2.254
XP_003459588.2	complement C1q tumor necrosis factor-related protein 3	0.389	0.643
XP_003444152.1	type-4 ice-structuring protein	0.5765	0.948
XP_019213314.1	hemoglobin subunit beta-A	0.3215	0.4815
XP_025755569.1	uncharacterized protein LOC100702986	2.3545	3.593
XP_003450540.1	apolipoprotein A-I	0.668	1.04
XP_025755734.1	serotransferrin-like isoform X1	0.7645	1.1855
NP_001298250.1	serotransferrin-like precursor	0.662	1.0275

## Abbreviations

WBC	white blood cell
MON	monocytes
NEU	neutrophil
DEGs	differential expressed genes
DEPs	differential expressed proteins
GBS	*Streptococcus agalactiae*
PTS	phosphotransferase system
